# The Application of the Teaching Games for Understanding in Physical Education. Systematic Review of the Last Six Years

**DOI:** 10.3390/ijerph17093330

**Published:** 2020-05-11

**Authors:** Raúl A. Barba-Martín, Daniel Bores-García, David Hortigüela-Alcalá, Gustavo González-Calvo

**Affiliations:** 1 Department of Didactics of Musical, Artistic and Body Expression, Faculty of Education of Segovia, University of Valladolid, 40011 Segovia, Spain; raulalberto.barba@uva.es; 2 Department of Physical Therapy, Occupational Therapy, Rehabilitation and Physical Medicine, Faculty of Health Sciences, Rey Juan Carlos University, 28922 Alcorcón, Madrid, Spain; daniel.bores@urjc.es; 3 Department of Specific Didactics, Faculty of Education, University of Burgos, 09001 Burgos, Spain; 4 Department of Didactics of Musical, Artistic and Body Expression, Faculty of Education of Palencia, University of Valladolid, 34004 Palencia, Spain; gustavo.gonzalez@uva.es

**Keywords:** Teaching Games for Understanding, pedagogical models, systematic review, educational research

## Abstract

A systematic review of the research conducted on Teaching Games for Understanding in Physical Education in the last six years (2014–2019), updating and expanding with new categories the last published review by Harvey and Jarret in 2014. Four databases were used to select those articles that included information on the implementation of Teaching Games for Understanding in different educational stages. According to PRISMA guidelines and including the PICO strategy after the exclusion criteria, 12 articles were fully assessed based on eight criteria: (1) year and author; (2) country; (3) number of participants, educational level, and duration of implementation; (4) type of research; (5) curricular content; (6) purpose of the research; (7) most relevant results; and (8) learning environment. The results showed how research focuses on both primary and secondary education, primarily in short-term interventions. Quantitative, qualitative and mixed research is used almost equally, and dealt evenly with sports and games, leaving motor skills, physical abilities and body expression underrepresented. Regarding the goals of the studies, motor and cognitive learning were the most frequently assessed, focusing on improvement of game development, such as tactical aspects, decision-making, technical skills or level of physical activity. The implementation of the model is carried out in too short a time to achieve significant outcomes. This review can help researchers and practitioners conduct Teaching Games for Understanding intervention programs in primary and secondary Physical Education. They must be rigorous when they claim that they implement this pedagogical model in schools.

## 1. Introduction

Sports have long occupied an important place in the Physical Education curriculum [1,2]. Its teaching through different models has been based, generally, on a sequencing or partitioning through games. These games can be of various types, e.g., cooperative, competitive, territorial, modified, etc., whose purpose is to work on one or several specific parts of the sport through rules, in order to get closer to a complete learning of the sport.

The technical method has traditionally been predominant in the teaching of physical activities and sports in Physical Education (PE) [3]. Through this form of teaching the teacher plans a sequence of prescriptive exercises, which are based on simulations of a part of the game, and leads them with a direct command [4]. The different tasks have a specific objective, usually in line with the development of some technical skill inherent to the game. This approach, therefore, assumes that a certain degree of skill must be acquired before an activity can be performed [5]. However, the demands of mastering a game require much more than just physical or technical skill [6,7]. We found that among the main criticisms that this method has received over the years is that it is incapable of serving all students, since it can only be developed effectively with the most skillful students; there is an excessive dependence on the instructions provided by the teacher, which limits the acquisition of autonomy of choice by students during the game, and, above all, the resulting learning is decontextualized from the real game, which makes it difficult to make decisions during the practice of the game [8,9].

Teachers and researchers in the field of PE have been working for years to bring other approaches to sports education. Based on the ideas of pedagogical models [10,11], there has been a strong development of these new proposals in recent years. However, their origin takes place in the 1970s, when proposals focused on tactics began to emerge [12]. These alternative models focus on the cognitive development and skills of students through decision-making during the game [13,14]. Learning is no longer focused on overcoming a specific objective, but rather on the resolution of problems that occur during a real game situation by students [15]. It was from these ideas that the Teaching Games for Understanding (TGfU) model was created.

## 2. Teaching Games for Understanding: From a Major Shift to the Need to Keep Moving Forward

The TGfU model emerged in the 1980s [16] as an alternative for teaching and learning sports games in PE. The appearance of this model generated great interest and represented an important revolution in the way sports were worked out from an educational perspective [17]. The understanding of sport became the focus of learning, and students were asked to consider the “why” of doing something during the development of the game, rather than the “how” [18]. The use of the body ceased to be instrumental and became connected to thought, context and the collective [19]. Development through the TGfU model was based on a combination of tactics and skills in real or simulated game contexts, with the aim of influencing improved decision-making and problem-solving during the game. The teacher also stopped playing a main role during the activities and now focused on making the students reflect through questions in order to make them acquire a tactical awareness. In this way, the TGfU model allowed for a global understanding of the students’ game by simultaneously providing cognitive, affective, social and physical learning [20], in addition to being beneficial for developing correct physical literacy in the student body [21].

The TGfU is based on four pedagogical principles [22]. These principles are: (1) transfer, which is achieved through the use of the global game, finding the tactical aspects common to the different sports; (2) modification-representation, consisting of the adaptation of the games to the age or skill level of the student body, preserving the tactical structure; (3) modification-exaggeration—this principle raises the possibility of including new rules or modifying them to help assimilate the main tactical contents; and (4) tactical complexity, where the tasks posed must be based on a progression in tactical difficulty.

The purpose of the TGfU, therefore, was to establish a model for developing sport through logical sequencing in learning. The experience of the students would no longer be based on a summary of games, but on the fact that these would serve to provide the students with a progression and balance [23]. To achieve this, it was considered important to establish common guidelines among sports, which would help to create categories of games through their similarities. Thus, classifications were developed that grouped them, mainly into four [24,25]: (1) target games, consisting of reaching a target with a mobile phone in the least number of attempts; (2) court or net/wall games—games in which a mobile phone is thrown within limits with the intention that it is returned by the opponent as late as possible; (3) field or striking/fielding—each team has its own zone and must send a mobile to the opponent, trying to make it difficult to return it; and (4) territory or invasion games—teams share the same space and defend and attack a target. In this way, teachers can help students to compare more easily the similarities of apparently different games, and then extrapolate them between sports such as basketball and football which are both territorial or invasion games.

Thorpe, Bunker and Almond [26] also proposed a series of stages for the development of the model. Through these procedures, they tried to explain, step by step, how teachers could help students to go through different levels in order to achieve a deep understanding of a game. These stages are: (1) students must first be able to understand the game form. For this, it is necessary, in the early years of school, to introduce children to a variety of game forms in accordance with their age and experience; (2) gradually, the students should learn to appreciate the game. From the outset children should understand the roles of the game that muse be played, no matter how simple they may be; (3) once they understand the rules, it is important that students acquire a tactical awareness—ways and means of creating space and denying space must be found to overcome the opposition. Decision-making: there is a difference between decisions based on “what to do” and “how to do it”, which allow both the student and the teacher to recognize and attribute decision-making deficiencies; (4) execution of skills is also important, but should always be seen in the context of the learner and the game; and finally, (5) when students are ready they should be able to execute actions in the context of the game. This is the observed outcome of the previous processes measured against criteria that are independent of the learner. It should be a measure of appropriateness of response as well as efficiency of technique.

All these considerations have been analyzed, criticized and extended by different researchers. When they proposed the model, these authors were aware of the need for future research to deepen the subject [27]. However, as stated by Kirk and MacPhail [28], it was not until early 2000 that there was an attempt to revise the model. It was these authors themselves who conducted in-depth research on the model and the latest publications and outlined a new version of the TGfU through situated learning. Their idea was to make it easier for teachers to implement the model and thus to direct it towards their understanding as a pedagogical model [29]. Holt, Strean, and Garcia-Bengoechea [30] also reformulated and expanded the initial TGfU model in an attempt to better address the affective domain. However, during these years there have also been misinterpretations of the model that have led authors, supported by the original creators of the model, to publish clarifications and to deepen their original ideas [17,31]. All of this has been done with the aim of facilitating the implementation of the TGfU model in schools and, thus, increasing research into the results achieved.

Thus, the main objective of this study was to review the scientific literature published in the last six years on the implementation of the TGfU in the school context, updating and expanding with new categories the last published review [1] to help teachers and researchers.

## 3. Materials and Methods

### 3.1. Search Sources

A systematic review of the literature published in the last six years on the implementation of the Teaching Games for Understanding model in the school environment was carried out. In order to find existing publications between January 2014 and December 2019, a search was initiated in four electronic databases: Taylor and Francis, ERIC, SCOPUS and Web of Science. The descriptors “Teaching Games for Understanding” and “TGfU” were used with the search operator OR.

### 3.2. Exclusion Criteria

The exclusion criteria used were as follows: (1) Duplicated articles; (2) articles not published in journals indexed in the Journal Citation Report (JCR) or the Scimago Journal Rank (SJR); (3) articles in languages other than English or Spanish; (4) articles in which the TGfU was not implemented in schools; (5) articles that did not explicitly allude to the TGfU, but rather to the use of games with methodologies that did not fit the basic characteristics of the model [16,22]; and (6) articles in which the TGfU was hybridized with other pedagogical models.

### 3.3. Limits and Methodology of the Search

The search was conducted following the guidelines of the Preferred Reporting Items for Systematic Reviews and Meta-Analyses (PRISMA) [32], including the PICO strategy: participants (e.g., primary, secondary, country), intervention (e.g., units, lessons, type of research (quantitative, qualitative, or mixed), comparators (e.g., Teaching Games for Understanding, TGfU), and outcomes (e.g., cognitive, affective, motor). The search ended on 20 March 2020.

### 3.4. Procedure

The research began in April 2019 and ended in March 2020. The first step was to establish the criteria for choosing articles, the exclusion criteria and the databases in which to carry out the search. With regard to the inclusion criteria, we checked in an initial search that the authors, regardless of language and context, used “Teaching Games for Understanding” and/or “TGfU” interchangeably. Therefore, these two terms were selected for the search. The researchers discussed the possibility of including the term “Physical Education”, to limit the search directly to the school and subject context. However, we considered that the inclusion of this term could bias the search too much and, finally, we decided to include this aspect as an exclusion criterion and analyze it through the reading of the articles. We then selected the exclusion criteria presented above. We took into account what was used by other authors in other predecessor systematic reviews and the aim of the research, related to the TGfU model and its implementation in schools. Once the inclusion and exclusion criteria had been defined, we established the databases in which to carry out the search. It was decided to select four of these databases. These databases were ERIC, Scopus, Taylor and Francis and Web of Science (WoS). We chose ERIC because it is the largest online database in the education field. We chose Scopus and WoS because they are the two most important citation databases in the world. They have great prestige at an international level and their growth in recent years, through the indexing of new journals and through the creation of new collections such as the Emerging Source Citation Index (ESCI), has allowed their indexed literature to increase. Taylor and Francis was selected because of its large number of journals—over 2600—and its international character, having a presence in all major geographies.

**Figure 1:**
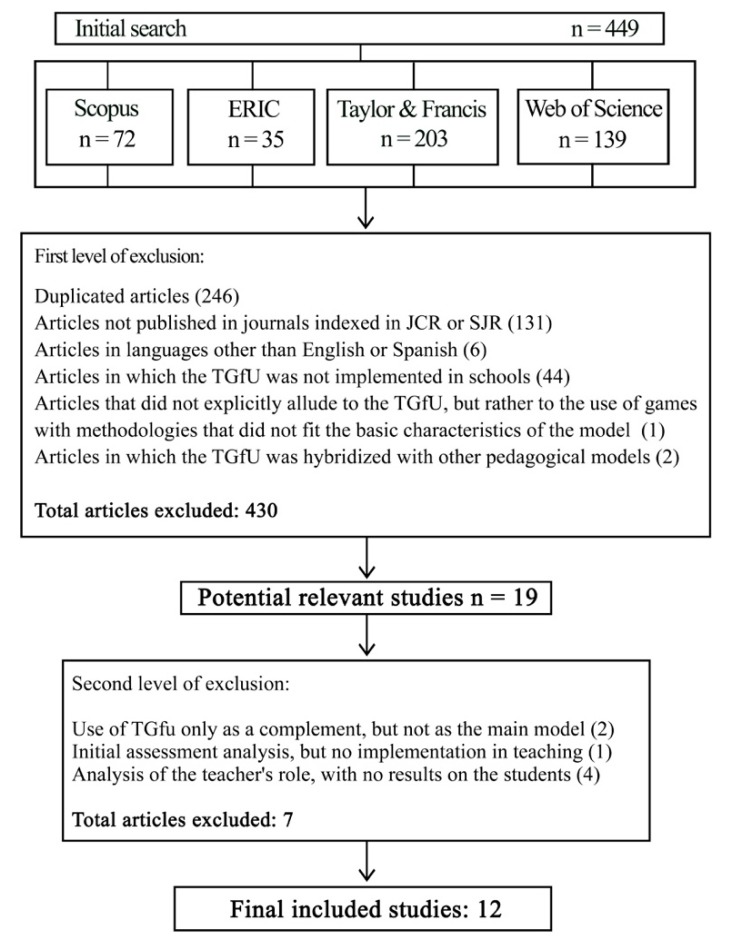
Flow diagram of the systematic search process.

All articles were extracted from the databases and analyzed through the MENDELEY (Elsevier, Comaland, USA). With the inclusion criteria, 449 publications were initially found using the mentioned descriptors: Taylor and Francis: 203 articles; ERIC: 35 articles; SCOPUS: 72 articles; Web of Science: 139 articles (Figure 1). Two researchers analyzed the articles individually, following the exclusion criteria, and pooled our results. Only 12 articles remained. Most of the discarded articles did not deal with the implementation of TGfU in schools, with extra-curricular football being the main context. Nine articles created doubts as to whether or not they belonged to the research when applying the exclusion criteria. These articles were analyzed individually and in depth by four researchers, after which it was determined that two of them were included in the review and seven others were not, for the following reasons: two analyzed another model and used TGfU as a complement, (one analyzed the use of video-guided debates and another analyzed the use of game-centered approaches), one analyzed an initial assessment and then implemented comprehensive learning models, such as TGfU and four analyzed the role of the teacher, but did not present learning outcomes for students. Finally, the four researchers conducted an individual analysis of the quality of the selected articles and shared it (Table 1). The aim of carrying out the whole process in a duplicate or parallel way among the researchers was to minimize the bias around the application of the exclusion criteria and the selection of articles. Broadly defined criteria were established among all the authors from the beginning of the research and the analysis processes were carried out, at least in duplicate, to try to minimize it.

Table 2 was constructed with the 12 final articles selected, after a thorough and systematic review process, where each one was described based on the following categories: (1) Author and year of publication: this field provides information on the authorship and distribution of the research in the last six years; (2) Country of application of the model: provides information on the countries in which the research has been carried out; (3) Number of participants, age and duration of experience: this category includes information on the variability of the sample used, both in the number of participants and in the level of education, as well as the duration of implementation; (4) Type of research details whether the study used quantitative, qualitative or mixed methods, as well as the main instruments; (5) Content: provides information on the curricular content worked on in the research through the TGfU; (6) Purpose: the objective/s of the study; (7) Results: the main results are presented, their contributions to the literature and the possibilities for replication; and (8) Learning outcome: information on the impact of the application in the different learning domains (affective, social, motor and/or cognitive).

## 4. Results and Discussion

The 12 articles selected between January 2014 and December 2019 are discussed around the eight elements used in the categorization set out in Table 2. The year is not included in the discussion as they are all from the last six years. The purpose and the results obtained have been grouped in the same section, due to the relationship between both categories.

### 4.1. Country

The results show a variety of countries in which school experiences using the TGfU model have been applied. The main place is Spain, where almost half of the articles analyzed are recorded—5 out of 12. The rest of the articles are widely distributed geographically, although there are other places where more than one implementation of the model has been carried out, with published results; this is the case with Canada and Malaysia, with two articles each. The remaining articles are distributed between different countries: one in Greece, one in the Netherlands and one in China. These results show how the model has expanded in contexts where until a few years ago there were hardly any implementations, as is the case with Canada and Malaysia, although in the study we have found two cases in each country. In 2008, Butler, Oslin, Mitchell and Griffin [33] asked Canadian physical education teachers to join the implementation of the TGfU model in schools, as was happening in other countries. On the other hand, Nathan [34] pointed out that in Malaysia, the TGfU was still in an early stage of implementation. The small number of implementations found does not allow us to make generalizations about the expansion of the model, although it does allow us to get an initial view about its settlement in certain areas and its adaptability to the curriculum of different countries, facts that will help us to establish correlations below.

### 4.2. Number of Participants, Educational Stage and Duration

The number of participants varies quite a lot among the research analyzed. The minimum number found is established by the research of Bracco et al. [35] with six students. These authors carried out an implementation of the TGfU, as in the rest of the research, however, for their study they only present the data of a very specific group within a class, since they are adolescent girls identified as disconnected from PE. In general, we find values that move between 21 and 41 students. There are five investigations that include between 71 and 448 students. These numbers are due to the fact that most of the research has been carried out in parallel in more than one class group or, at least, dividing the same class group into two halves, in such a way that each subgroup contributed a significant number of students. In this way, the TGfU model could be compared between different educational levels [36], with the implementation of other models, generally the technical one [34,37], or even differentiating the extracurricular experience that the students had prior to the activity [38]. In some cases, the distinction has been made on the basis of gender [39,40], including the study by Bracco et al. [35], which focused only on girls. In this sense, the use of popular sports or invasion games in PE through technical models and without specific teaching intervention, have been shown to be empowering for inequalities [41,42,43]. Therefore, the comparative analysis of all these factors is important to understand in depth the results that the application of the TGfU has on students, according to their characteristics, skills, contexts or sex, and thus achieve the transformation of the models that do not.

As for the educational stage in which the research is implemented, there is also great variety. Since the studies are carried out in different countries, for this part of the analysis we have grouped them by age and by the general levels that all education systems fulfill, differentiating between primary education (5–12 years) and secondary education (13–18 years). The number of studies is distributed practically equally between these two large groups. Studies with a greater number of participants carried out implementation at various educational stages, while studies with a smaller number carried out implementation at the same grade or course. In these latter studies, it is worth noting that, within those carried out in primary education, most of them occur at the highest ages (11–12 years). As for secondary education, the participants in the studies do not exceed 16 years of age. The implementation of the TGfU at these ages can be significant in creating positive experiences in students that can be transferred later to their daily lives and thus reduce the abandonment of physical activity that occurs when they reach adolescence [44]. As play is an important method of learning at early ages and the benefits of this model for failing experimentation, decision making and learning occur from errors at such ages [45], it therefore seems important to increase the number of studies that implement this model from early stages, whose good experience could lead students to a better development of skills as well as intellectual and abstract thinking during their adolescence.

Finally, the duration of experiences indicate that they occur during a short or medium-short period. If we group the articles, we find that they are usually performed in three large blocks: (a) below 9 lessons; (b) between 12 and 16 lessons; and (c) above 18 lessons, all of them lasting between 40–60 min. In some cases, the total number of lessons includes an initial session and a final session, which serve as pre- and post-TGfU evaluations [38,46]. The first two blocks are where more research is recorded, which is consistent with the fact that all studies, except for Hortigüela-Alcalá and Hernando-Garijo [37] where an implementation was carried out in three consecutive units (24 lessons), were based on a single unit or part of it. However, when the study is carried out in short periods, below 9 sessions, most of the authors agree that this short time is a limitation of the study, since there are certain aspects, such as the learning of technical skills, which require a longer period of teaching-learning activities through the TGfU model [38]. The need for the TGfU to develop the previously explained factors in the students makes it necessary to intervene with time in order to verify the results obtained in learning from start to finish.

Another element of analysis is the teacher training component. As stated in the previous studies by Moy, Renshaw and Davids [47], teachers consider that before carrying out the TGfU model it is necessary to have a thorough knowledge of the model itself and of each of the disciplines in order to make the appropriate modifications during development. This fact is also reflected in some articles, when explaining how researchers invested some time in training teachers before the intervention e.g., [34,37,40], and the research lasted for months. For these authors, teacher training in the model is vital so that the implementation of the model is as real as possible, without running the risk of applying the model without respecting its essential characteristics.

### 4.3. Type of Research

Qualitative (25%), quantitative (41.6%) and mixed (33.4%) methods have been used. In the qualitative studies, discussion groups with the participants, individual interviews, observations and analysis of the students’ discourse were used. In the quantitative studies, protocols such as Think-Aloud validated tools such as the Game Performance Evaluation Tool (GPET) and performance measurement instruments such as Polar Team Pro TM or systematic observation sheets have been used. The mixed studies used the qualitative data collection techniques explained above and quantitative techniques in line with the previous ones such as: the questionnaire to measure motivational strategies in physical education lessons (QMSPE), the Game Performance Assessment Instrument or the Actigraph GT3X and AAHPERD-BST activity monitor. There is a trend towards the use of quantitative methods when what is sought is to know the effectiveness, physical performance, technical execution or skill level, while qualitative research focuses on knowledge of aspects such as motivation, relationships with previous knowledge or experience, collaborations between students or future adherence to physical activity. Therefore, while quantitative approaches allow us to understand the improvements in skills associated with the development of play through TGfU, qualitative methods bring a pedagogical approach to the model, trying to connect students with the previous reasons that lead them to their skillful development and their interest in participating and practicing the games, through the meaning they give to their experiences. In both cases, there is a great contribution to the deep understanding of the TGfU model, its advantages and the main aspects it affects; this is what is really important when establishing a research design around pedagogical models in PE [48].

### 4.4. Content

With regard to the topics addressed, some studies show similarities between them, although within a wide variety. All the research is directed at a single content, except the one by Hortigüela-Alcalá and Hernando-Garijo [37], which deals with the teaching of three different sports modalities. This is in line with the analysis made earlier about the number of teaching units in which the model was implemented. Basketball is the most represented content, since it appears in three researches; in some, as in Wang and Wang’s [40], this is chosen by the students themselves.

There are coincidences in the relationship between the content and the country. None of the research developed in Canada refer to a specific content, but to the generic term “territorial games”, while the two studies from Malaysia focus on badminton. The rest of the research shows a great variety; in Spain there is also indoor soccer, floorball or handball; in Holland baseball, and in Greece volleyball. Although the number of studies from each country is small enough to establish causal relationships, by doing a deeper analysis we can know how these contents usually correspond to the popular or more successful sports in each context. Thus, for example, Malaysia is one of the countries with the most tradition in modern and organized badminton [49], while the Netherlands has one of the most successful baseball teams at the international level.

This analysis shows how the TGfU is a model that is transferable to different sports [50,51,52], which makes us understand that their international expansion is great, as they are able to be used in the learning of the most representative and developed sports by the students of each place. Moreover, this is an important advantage, since it directly influences the transferability of the use of this model in PE to the extracurricular development of the students.

### 4.5. Purpose and Results

The heterogeneity analyzed so far is also reflected in the multiple objectives and main results of the studies. Most of the researchers focused their objectives on aspects intrinsic to the improvement of game development, such as tactical aspects, decision making, technical skills, performance or physical activity level [53]. Generally, these studies provide positive results about how the use of the TGfU model leads to an improvement in some specific aspect of student performance and understanding of the game. Other articles focus on more pedagogical aspects such as motivation, participation or established relationships [54]. These articles follow the line of research such as that of Coulter and Ni Chroinin [55], by showing in their results how pedagogical models focused on student learning, such as the TGfU, are more positive for their development in PE. Some of the articles analyzed combine both purposes [56].

In addition, we find several studies that compare the results obtained through the application of the TGfU model with other forms of learning, such as: the technical model [34,37], prescriptive feedback [46] or other comprehensive models, such as the Contextualized Sports Literacy Model (CSAM) [35]. They are even compared from a multidisciplinary perspective [57]. The results of these studies allow us to analyze the potential of TGfU in contrast to other models or forms of learning, but they are also a source for understanding the weaknesses of the model, such as the greater difficulty of contributing to the social domain than other models such as Cooperative Learning or Sport Education.

In this sense, as in previous research, the results of the different articles highlight the TGfU as a positive pedagogical model in PE by promoting learning to play through understanding and knowledge, thus achieving an intrinsic motivation in students [58], which helps them to acquire good exercise habits and enjoy the fun of playing [59]. However, they also expose the weaknesses of this model and open the door to new concepts, modifications or hybrids that manage to solve them, as proposed by Kirk and MacPhail [28].

### 4.6. Learning Outcomes

In this last section we have analyzed the main domains developed in each piece of research, understanding them as the elements into which the type of learning acquired after a teaching-learning process can be divided [9]. These domains are: cognitive, motor, social and affective; all four of these are suitable for teaching through the TGfU model [20].

The development of the motor and cognitive domains have been the most frequent learning outcomes in the research, which is in line with the foundations of the model established by Bunker and Thorpe [16]. These authors developed the TGfU model with the proposal of using games through understanding, developing tactical knowledge and improving problem solving through skill execution and decision making. Cognitive development is the main domain in the research of Chatzipanteli et al. [53], Gil et al. [46] and Slater and Butler [57], paying special attention to the knowledge acquired by students. For its part, motor development stands out in the studies of González-Víllora [36] and Wang and Wang [40], which provide results about physical and physiological performance, as well as levels of physical activity.

The affective domain is also present in several investigations. This is a product of the influences of sports psychology and motivation towards physical activity on which the TGfU model was based [60]. Studies show how using TGfU also leads to learning that is directed towards student motivation, participation or the development of positive attitudes [34,37,56]. This domain only appears as the main one in the research of Bracco et al. [35].

Finally, the social domain hardly appears in the publications analyzed. It is only found in the research of Koekoek and Knoppers [54]. The TGfU model focuses on the development of the game, but does not pay attention to the relationships that are established, since even in six of the investigations the results are not particularly positive and show how, although sometimes students consider collaboration to be necessary, they also value it as a source of distraction. As a result, hybrid models have emerged that encourage more work in the social domain, such as those that unite TGfU and cooperative learning [61,62].

## 5. Conclusions

The present systematic review on TGfU shows an in-depth analysis and correlations between the main features of research on TGfU implementation in schools in recent years. Among the aspects found there is a difference between the dissemination of the method and its application in school contexts, since we found a high number of publications related to the study topic, but we have only found 12 articles in the last six years focused on implementations of the model in the school context. This indicates a great contrast between theoretical research or research outside school contexts and those carried out in schools and during PE. This circumstance is revealing for PE teachers and researchers, as it shows the need to continue addressing the subject through an understanding of the real possibilities of the TGfU in the subject. The results show that these studies can be approached from different methods (qualitative, quantitative and mixed) and instruments, although each one of them seems to be able to address some learning areas or contents more effectively than others. The domains, motor and cognitive, were the most analyzed, in contrast to the affective and social domains, despite their importance in the educational field. Once again, the results open up an interesting avenue of development for future researchers. It is necessary to understand that research on the implementation of the TGfU needs a long time, as the results analyzed show us and, in most cases, in addition to the implementation and data collection, it is necessary to invest time in the previous preparation of teachers since they must master both the content and the characteristics of the model [63,64]. Perhaps these are some of the consequences of the small number of studies found, despite this being a model with broad benefits for students; its solution could be found in establishing their learning in the university programs of initial teacher training [65].

The main contribution of the study has been to update the literature on the implementation of the TGfU from 2014. This article provides a comprehensive review of the research that has implemented the TGfU model in the educational context over the past six years. This is a review that has not been done before, although there are other previous reviews that are introduced in the existing literature on TGfU, but they do so from an analysis based on game-centered approaches (GCA) [1,34] and not necessarily on school PE. Oslin and Mitchell carried out their review up to 2006 around five common objectives of physical education and sport programs [66], while Harvey and Jarrett did so from then until 2013 following the guidelines and recommendations set out for future studies by their predecessors [1]. Thus, our research addresses a period of time in which no one has conducted a systematic review and, moreover, focuses specifically on the TGfU model [16]. Regarding the method and categories evaluated, we have followed the procedure used in other current systematic reviews on pedagogical models such as Bores-García, Hortigüela-Alcalá, Fernandez-Río, González-Calvo, and Barba-Martín [67].

We understand that the study has limitations associated with the results obtained and the level of review, such as the risk of bias, incomplete recovery of identified research or information bias. A rigorous process was undertaken to try to minimize these limitations which has been explained in detail in the article so that the reader can understand it. As for future research, it will be of interest to address the study of emerging hybrid models, in particular the TGfU, with other models, as well as to analyze extracurricular contexts. These will make it possible to extrapolate knowledge into an implementation in school practices. This article may be of particular interest to teachers interested in improving their teaching practice and implementing understanding-based game-learning models such as the TGfU, with an emphasis on the model’s approach to its application in the classroom. It could also be of interest for the institutional and scientific advancement of PE, by providing a vision of the importance of the union of cognitive and motor development for the development of skills and deep understanding of games.

## Figures and Tables

**Table 1:** Investigation quality score checklist.

Research	Program Description	JCR/SJR Inclusion	Methodology	Sample	Length	Total Score	Quality Level
Bracco et al. (2019)	2	1	2	1	0	6	MQS
Chatzipanteli et al. (2016)	2	2	2	2	1	9	HQS
Gil et al. (2019)	2	2	2	1	2	9	HQS
González-Víllora et al. (2019)	1	2	2	2	1	8	HQS
Hortigüela-Alcalá & Hernando-Garijo (2017)	2	2	2	2	2	10	HQS
Koekoek & Knoppers (2015)	2	2	1	1	2	9	HQS
Morales-Belando et al. (2018)	1	2	2	1	0	6	MQS
Nathan (2016)	2	1	2	1	1	7	HQS
Pizarro et al. (2016)	2	2	2	1	1	8	HQS
Shahril et al. (2017)	2	2	2	2	1	9	HQS
Slater & Butler (2015)	1	2	1	1	1	6	MQS
Wang & Wang (2018)	2	2	2	2	1	9	HQS

Note: program description (did the research offer a detailed description of the program?): ‘0′: not included, ‘1′: brief and undetailed description, and ‘2′: detailed description; JCR/SJR inclusion (was the study published in a journal indexed on the JCR or SJR?): ‘0′: not indexed, ‘1′: indexed on SJR, and ‘2′: indexed on JCR; methodology (did the paper report in detail the methodological process used?): ‘0′: not reported, ‘1′: reported but imprecise (not completely), and ‘2′: exhaustive description reported; sample (number of participants): ‘0′: fewer than 10 participants, ‘1′: from 10 to 50 participants, and ‘2′: more than 50 participants; length (duration): ‘0′: less than eight lessons, ‘1′: from nine to 14 lessons, and ‘2′: more than 15 lessons; JCR: Journal Citation Report; SJR: Scimago Journal Rank; HQS: high quality study (7–10), MQS: moderate quality study (5–7).

**Table 2:** Summary of Teaching Games for Understanding (TGfU) articles published between 2014 and 2019.

Author and Year	Country	Number of Participants, Grade and Duration	Type of Research	Content	Purpose	Results	Learning Outcomes
Bracco, Lodewyk & Morrison (2019)	Canada	6 female students. Two weeks (6 lessons)	Qualitative: observation field notes, focus group interviews (before and after the unit) and individual interviews (after the unit).	Territorial games	Analyze how the TFGU could support PE participation of six adolescent girls who were identified as being disconnected from PE.	Students experienced increased participation and effort, learning, affection and motivation in the TGfU game unit. TGfU was beneficial because of its student- and game-centered nature; however, students also had some reservations (time and sports options). Ultimately, TGfU can support girls’ participation in physical education by engaging them and encouraging them to increase their participation in a holistic manner.	Affective
Chatzipanteli, Digelidis, Karatzoglidis & Dean (2016)	Greece	71 students aged 11–12. 1 unit	Quantitative: Think-Aloud protocol before and after the investment	Volleyball	To examine the effectiveness of TGfU in developing metacognitive behavior in elementary students.	The use of tactical models facilitates the development of metacognitive behavior in primary students.	Cognitive
Gil, del Villar-Álvarez, Práxedes-Pizarro & Moreno-Domínguez, (2019)	Spain	37 students from two different groups in the 6th year of a primary school, aged between 11 and 12. The experimental group consisted of 20 students and the control group of 17 students. 18 PE lessons.	Quantitative: systematic indirect and external observation of decision-making.	Basketball	To analyze the effect of a comprehensive, question-based teaching program for improving passing and shooting decisions in a basketball unit in PE.	The results obtained showed that, after the intervention, the students who received the questionnaire when developing training activities improved their decision making compared to those who did not.	Cognitive
González-Víllora, Sierra-Díaz, Pastor-Vicedo & Contreras-Jordán (2019)	Spain	112 students from first to sixth grade. 12 lessons of 135 min per week.	Quantitative: quasi-experimental and cross-sectional study with pre- and post-test evaluations.	Futsal	To compare the degree of physical and physiological performance in various indoor football games that have been implemented through two MsBPs: the Teaching games for Understanding (TGfU) and the Contextualized Sports Literacy Model (CSAM). Analyze the relationship between physical and physiological variables.	The results showed significant differences in the physical and physiological variables in the GCS implemented in the CSAM over the games implemented during the TGfU. In addition, multilevel and MANCOVA post-test analyses show significant differences in physical and physiological performance during 4 vs 4 post-test SSCG in CSAM students, in contrast to TGfU students (*p* < 0.001).	Motor
Hortigüela-Alcalá & Hernando- Garijo (2017)	Spain	237 students in the study (58.3% men, 41.7% women), divided into 1st, 2nd, 3rd and 4th grade, and two teachers. 24 lessons of three sports units. Each unit 8 lessons.	Mixed, quasi-experimental. Quantitative: QMSPE. Qualitative: semi-structured interview of the two teachers.	Basketball, floorball and handball.	1) to evaluate the impact of TGfU on student motivation and performance in sport; 2) to study how variables such as grades, academic results, and extracurricular sports practice influence student motivation and interest in sport; and 3) to contrast PE teachers’ perceptions of the importance of methodology for teaching sport.	The results revealed that the group that used TGfU showed greater motivation and achievement in PD than the control group. Significant differences in achievement were found. The participants with better academic results in the group that used TGfU were more positive in sports participation. Meanwhile, students who played more extracurricular sports in the control group were more actively involved in sports.	Motor and affective
Koekoek & Annelies Knoppers (2015)	The Netherlands	25 students aged 12–13 (1st year of secondary school). 1 unit.	Qualitative: eight discussion groups with participating students.	Baseball	Explore the perceptions of collaboration, group formation and friendship in a baseball unit.	The presence of playmates generates different interpretations: for some they are necessary collaborators, for others they are a distraction and for others they are a source of criticism.	Social
Morales-Belando, Calderón & Arias-Estero (2018)	Spain	41 students (23 boys and 18 girls). 6th Grade Primary School. Unit of 8 lessons	Mixed: pre-test and post-test, Game Performance Assessment Instrument, two psychological scales and two discussion groups with students and teacher.	Floorball	To check if students improved in variables related to performance and adherence after a TGfU-based unit.	Improvement in decision making, technical execution, support, game performance, enjoyment, participation in the game, perceived competence and decision to remain physically active after the unit.	Motor, cognitive and affective
Nathan, S (2016)	Malaysia	32 students of 15 years old with equal numbers of males and females. 5 weeks, 12 lessons.	Mixed: Quantitative Data: Observation instrument to examine the skill components and cognitive decision making of game performance Qualitative Data: Recording of teacher reflections and observations.	Badminton	To examine the effects that a revised model of TGfU compared to Skill Drill Technical (SDT) had on learning movement skills in badminton, including returning to base, making decisions, and executing skills while performing a doubles game. Explore teachers’ perceptions of navigation between the two models	The results indicated for the movement to the base in the doubles game indicate a significant improvement, after the intervention through TGfU. Regarding decision making and execution of skills in the game of doubles, the analysis revealed no significant differences after the intervention. Findings from teacher reflection indicated the importance of mini-game in TGfU and SDT models, as students enjoyed, and developed positive attitudes to win or lose in game situations.	Motor, cognitive and affective
Pizarro, García-González, Cortés, Moreno-Arroyo, Domínguez (2016)	Spain	21 students from two different groups of 1st Secondary. 2 evaluation lessons (one of initial evaluation and another of final evaluation) and 7 of development or learning, being the unit composed by nine lessons.	Quantitative: “Game Performance Evaluation Tool (GPET)” observation instrument.	Futsal	To analyze the effect of a Comprehensive Teaching programme on decision making and execution of passing and driving in futsal in an educational context.	The results show a significant improvement in decision making for approval after the application of the program to the inexperienced group; however, these differences were not found in the experienced group. With regard to implementation, the programme did not affect either group positively or significantly in this variable in any of the actions studied.	Cognitive and motor
Shahril, Jani & Salimin (2017)	Malaysia	448 student samples	Quantitative. The study design is descriptive and pre-experimental.	Badminton	Identify the learning level of students through the Performance Assessment Instrument (PAI) model for the cognitive, psychomotor and affective domains in badminton games based on the TGfU method. Compare the level of learning of male and female students in general	Overall, the percentage of student learning is 79.65% and a few students who reach level 4 can master strategies, tactics and organize values throughout the game. The study also found that there is no significant difference (p = 0.222) in students’ average performance levels by gender. Analysis of the data shows that the average performance of men is higher than the average score of women. The level of agreement among teachers on the use of the PAI model is excellent, with 83.71% of teachers agreeing with the model.	Cognitive, motor and affective
Slater & Butler (2015)	Canada	A sixth grade class of 30 students approximately 11 years old. Approximately one month for three 40–60 min class periods per week.	Qualitative: Discourse analysis.	Territorial games	Compare the knowledge structures in science language and the language of a teaching unit on inventing territory games that was developed and taught to a sixth grade physical education class using a TGfU approach.	The results suggest that in the discourse of the physical education and science classes, the six Knowledge Structures identified by Mohan as comprising a framework for activities (KF) appear in similar patterns.	Cognitive
Wang & Wang (2018)	China	A total of 118 students in four classes (two TGfU groups and two technique groups). 6 weeks, 12 lessons (2 lessons per week).	Mixed: quantitative data using Actigraph GT3X and AAHPERD-BST Activity Monitor. Qualitative data by interview.	Basketball	To investigate the effectiveness of the TGfU intervention on the moderate to vigorous physical activity levels of students in grades 9 and 10. Analyze how gender and ability levels influence MVPA levels during TGfU. The third purpose of this study is to explore the factors that determine students’ MVPA levels during TGfU classes by conducting interviews with teachers and students.	The results reveal that the TGfU and the technical group exhibited significantly improved MVPA levels in the intervention phase. During the intervention period, the MVPA time of the TGfU group was significantly higher than that of the technical group. In addition, in the TGfU classes, boys spent significantly more time participating in MVPA than girls. However, no significant differences were determined between the MVPA levels of high- and low-grade students.	Motor

## References

[1] Harvey S., Jarrett K. (2014). A review of the game-centred approaches to teaching and coaching literature since 2006. Phys. Educ. Sport Pedag..

[2] Smith M.A., St Pierre P.E. (2009). Secondary Students’ Perceptions of Enjoyment in Physical Education: An American and English Perspective. Phys. Educ..

[3] Kagan S., Kagan M. (2009). Kagan Cooperative Learning.

[4] Raiola G., Tafuri D. (2015). Teaching method of physical education and sports by prescriptive or heuristic learning. J. Hum. Sport Exerc..

[5] Méndez A., Valero A., Casey A. (2010). What are we being told about how to teach games? A three-dimensional analysis of comparative research into different instructional studies in Physical Education and School Sports. Rev. Int. Med. Act. Fís. Deporte.

[6] Thorpe R., Bunker D. (1983). Issues that arise when preparing to teaching for understanding. Bull. Phys. Educ..

[7] Turner A., Martinek T.J. (1995). Teaching for Understanding: A Model for Improving Decision Making During Game Play. Quest.

[8] Hopper T. (2002). Teaching games for understanding: The importance of students emphasis over contents emphasis. J. Phys. Educ. Recreat. Dance.

[9] Kirk D. (2010). Physical Education Futures.

[10] Haerens L., Kirk D., Cardon G., De Bourdeaudhuij I. (2011). Toward the development of a pedagogical model for health-based physical education. Quest.

[11] Kirk D. (2013). Educational value and models-based practice in physical education. Educ. Theory Philos..

[12] Mahlo F. (1969). L’acte Tactique en Jeu.

[13] Abad M.T., Collado-Mateo D., Fernández-Espínola C., Castillo E., Giménez F.J. (2020). Effects of Teaching Games on Decision Making and Skill Execution: A Systematic Review and Meta-Analysis. Int. J. Environ. Res. Public Health.

[14] Tallir I., Lenoir M., Valcke M., Musch E. (2007). Do alternative instructional approaches result in different game performance learning outcomes? Authentic assessment in varying game conditions. Int. J. Sport Psychol..

[15] Singleton E. (2009). From command to constructivism: Canadian secondary school physical education curriculum and teaching games for understanding. Curric. Inq..

[16] Bunker D., Thorpe R. (1982). A model for the teaching of games in secondary schools. Bull. Phys. Educ..

[17] Harvey S., Pill S., Almond L. (2018). Old wine in new bottles: A response to claims that teaching games for understanding was not developed as a theoretically based pedagogical framework. Phys. Educ. Sport Pedag..

[18] Hopper T., Kruisselbrink D. (2001). Teaching Games for Understanding: What does it look like and how does it influence student skill acquisition and game performance?. J. Teach. Phys. Educ..

[19] Butler J. (2016). We Are What We Teach: TGfU as a Complex Ecological Situation. Res. Q. Exerc. Sport.

[20] Light R., Fawns R. (2003). Knowing the Game: Integrating Speech and Action in Games Teaching Through TGfU. Quest.

[21] Mandigo J., Lodewyk K., Tredway J. (2019). Examining the Impact of a Teaching Games for Understanding Approach on the Development of Physical Literacy Using the Passport for Life Assessment Tool. J. Teach. Phys. Educ..

[22] Thorpe R., Bunker D., Almond L., Pieron M., Graham G. (1984). A change in the focus of teaching games. Proceedings of the Sport Pedagogy: Olympic Scientific Congress.

[23] Werner P., Thorpe R., Bunker D. (1996). Teaching Games for Understanding: Evolution of a Model. J. Phys. Educ. Recreat. Dance.

[24] Ellis M. Similarities and differences in games: A system for classification. Proceedings of the AIESEP Conference.

[25] Werner P., Almond L. (1990). Models of games education. JOPERD.

[26] Thorpe R., Bunker D., Almond L. (1986). Rethinking Games Teaching.

[27] Thorpe R., Bunker D., Thorpe R., Bunker D., Almond L. (1986). Where are we now? A games education. Rethinking Games Teaching.

[28] Kirk D., MacPhail A. (2002). Teaching games for understanding and situated learning: Rethinking the Bunker-Thorpe model. J. Teach. Phys. Educ..

[29] Kirk D. (2017). Teaching games in physical education: Towards a pedagogical model. Rev. Port. Ciênc. Desporto.

[30] Holt N.L., Strean W.B., García-Bengoechea E. (2002). Expanding the teaching games for understanding model: New avenues for future research and practice. J. Teach. Phys. Educ..

[31] Butler J. (2014). TGfU Would you know if you saw it? Benchmarks from the tacit knowledge of the founders. Eur. Phys. Educ. Rev..

[32] Moher D., Liberati A., Tetzlaff J., Altman D.G., PRISMA Group (2009). Preferred reporting items for systematic reviews and meta-analyses: The PRISMA statement. PLoS Med..

[33] Butler J., Oslin J., Mitchell S., Griffin L. (2008). The Way Forward for TGfU: Filling the Chasm between Theory and Practice. Phys. Health Educ. J..

[34] Nathan S. (2016). Badminton instructional in Malaysian schools: A comparative analysis of TGfU and SDT pedagogical models. SpringerPlus.

[35] Bracco E., Lodewyk K., Morrison H. (2019). A case study of disengaged adolescent girls’ experiences with teaching games for understanding in physical education. Curric. Stud. Health Phys. Educ..

[36] González-Víllora S., Sierra-Díaz M.J., Pastor-Vicedo J.C., Contreras-Jordán O.R. (2019). The way to increase the motor and sport competence among children: The contextualized sport alphabetization model. Front. Physiol..

[37] Hortigüela-Alcalá D., Hernando-Garijo A. (2017). Teaching Games for Understanding: A Comprehensive Approach to Promote Student’s Motivation in Physical Education. J. Hum. Kinet..

[38] Pizarro A.P., García-González L., Cortés Á.M., Moreno-Arroyo M.P., Domínguez A.M. (2016). Aplicación de un programa de intervención para mejorar la comprensión táctica en fútbol sala: Un estudio en contexto educativo. Movimento.

[39] Shahril M.I., Jani J., Salimin N. (2017). Performance assessment instrument (PAI) model for badminton based on teaching games for understanding (TGfU). Adv. Sci. Lett..

[40] Wang M., Wang L. (2018). Teaching Games for Understanding Intervention to Promote Physical Activity among Secondary School Students. BioMed Res. Int..

[41] Gutierrez D., García-López L.M. (2011). Gender differences in game behaviour in invasion games. Phys. Educ. Sport Pedag..

[42] Mesquita I., Farias C., Hastie P. (2012). The impact of a hybrid sport-education-invasion games competence model soccer unit on students decision making skill execution and overall game performance. Eur. Phys. Educ. Rev..

[43] Stelzer J., Ernest J.M., Fenster J., Langford G. (2004). Attitudes toward physical education: A study of high school students from four countries: Austria, Czech Republic, England, and USA. Coll. Stud. J..

[44] Biddle S., Gorely T., Stensel D. (2004). Health-enhancing physical activity and sedentary behavior in children and adolescents. J. Sport Sci..

[45] Hodges-Kulinna P. (2008). Models for Curriculum and Pedagogy in Elementary School Physical Education. Elem. Sch. J..

[46] Gil V.M., del Villar-Álvarez F., Práxedes-Pizarro A., Moreno-Domínguez A. (2019). El cuestionamiento como herramienta fundamental para el desarrollo de la toma de decisiones de los alumnos en educación física. Movimento.

[47] Moy B., Renshaw I., Davids K. (2014). Variations in Acculturation and Australian Physical Education Teacher Education Students’ Receptiveness to an Alternative Pedagogical Approach to Games Teaching. Phys. Educ. Sport Pedag..

[48] Fletcher T., Ní Chróinín D., Price C., Francis N. (2018). Teacher educators’ enactment of pedagogies that prioritize learning about meaningful physical education. Curric. Stud. Health Phys. Educ..

[49] Lim P.H., Aman M.S. (2017). The History of Modern Organized Badminton and the Men’s Team Thomas Cup Tournaments, 1948–1979. Int. J. Hist. Sport.

[50] Fernández-Río J., Calderón A., Hortigüela-Alcalá D., Pérez-Pueyo A., Aznar-Cebamanos M. (2016). Modelos pedagógicos en Educación Física: Consideraciones teórico-prácticas para docentes. Rev. Esp. Educ. Fís. Deporte.

[51] Fernández-Río J., Hortigüela-Álcalá D., Pérez-Pueyo Á. (2018). Revisando los modelos pedagógicos en educación física. Ideas clave para incorporarlos al aula. Rev. Esp. Educ. Fís. Deporte.

[52] Pérez-Pueyo Á., Hortigüela-Alcalá D., Fernández-Río J. (2020). Evaluación formativa y modelos pedagógicos: Estilo actitudinal, aprendizaje cooperativo, modelo comprensivo y educación deportiva. Rev. Esp. Educ. Fís. Deporte.

[53] Chatzipanteli A., Digelidis N., Karatzoglidis C., Dean R. (2016). A tactical-game approach and enhancement of metacognitive behaviour in elementary school students. Phys. Educ. Sport Pedag..

[54] Koekoek J., Knoppers A. (2015). The role of perceptions of friendships and peers in learning skills in physical education. Phys. Educ. Sport Pedag..

[55] Coulter M., Ni Chroinin D. (2013). What is PE?. Sport Educ. Soc..

[56] Morales-Belando M.T., Calderón A., Arias-Estero J.L. (2018). Improvement in game performance and adherence after an aligned TGfU floorball unit in physical education. Phys. Educ. Sport Pedag..

[57] Slater T., Butler J.I. (2015). Examining connections between the physical and the mental in education: A linguistic analysis of PE teaching and learning. Linguist. Educ..

[58] Stolz S., Pill S. (2014). Teaching Games and Sport for Understanding: Exploring and Reconsidering its Relevance in Physical Education. Eur. Phys. Educ. Rev..

[59] Long W.X., Lung W.H. (2018). An Action Research: Teaching Games for Understanding on Badminton in a Junior School.

[60] Thorpe R., Williams T., Almond L., Sparkes A. (1992). The psychological factors underpinning the “teaching for understanding games” movement. Sport and Physical Activity: Moving toward Excellence.

[61] Chiva-Bartoll Ó., Salvador-García C., Ruiz-Montero P.J. (2018). Teaching games for understanding and cooperative learning: Can their hybridization increase motivational climate among physical education students?. Croat. J. Educ..

[62] Fernández-Rio J., Méndez-Giménez A. (2016). El aprendizaje cooperativo: Modelo pedagógico para educación física. Retos.

[63] Harvey C., Cushion C., Massa-González A. (2010). Learning a new method: Teaching Games for Understanding in the coaches’ eyes. Phys. Educ. Sport Pedag..

[64] Harvey S., Cushion C., Sammon P. (2015). Dilemmas faced by pre-service teachers when learning about and implementing a game-centred approach. Eur. Phys. Educ. Rev..

[65] Lodewyk K.R. (2015). Relations between Epistemic Beliefs and Instructional Approaches to Teaching Games in Prospective Physical Educators. Phys. Educ..

[66] Oslin J., Mitchell S., Kirk D., MacDonald D., O’Sullivan M. (2006). Game-Centred Approaches to Teaching Physical Education. The Handbook of Physical Education.

[67] Bores-García D., Hortigüela-Alcalá D., Fernández-Río J., González-Calvo G., Barba-Martín R.A. (2020). Research on Cooperative Learning in Physical Education. Systematic Review of the Last Five Years. Res. Q. Exerc. Sport.

